# The Impact of ‘Selfie’ Tourism on the Behaviour and Welfare of Brown-Throated Three-Toed Sloths

**DOI:** 10.3390/ani8110216

**Published:** 2018-11-19

**Authors:** Gemma Carder, Tinka Plese, Fernando Carniel Machado, Suzanne Paterson, Neil Matthews, Laura McAnea, Neil D’Cruze

**Affiliations:** 1 World Animal Protection, London WC1X 8HB, UK; icenando@gmail.com (F.C.M.); suzipaterson@hotmail.co.uk (S.P.); neilsmatt2@gmail.com (N.M.); lmcanea@gmail.com (L.M.); neildcruze@worldanimalprotection.org (N.D.); 2 Aiunau Foundation, Circular 1ª, No. 73—20. Medellín, Colombia; f.aiunau@gmail.com

**Keywords:** *Bradypus variegatus*, animal welfare, affective states, wildlife tourism, human-animal interactions

## Abstract

**Simple Summary:**

The use of wild animals as photo props is a potential animal welfare concern that is prevalent across Latin America. The present study documents animal welfare concerns associated with the use of wild caught brown-throated three-toed sloths (*Bradypus variegatus*) for wildlife ‘selfies’ by tourists at three locations in Manaus, Brazil and Puerto Alegria and Iquitos in Peru. Between 4 October 2016 and 8 April 2017 researchers attended 34 tours, where 17 sloths were observed. The sloths were; (1) held on average by 5 people (during each observation); (2) frequently exposed to physical manipulation of the head and/or limbs; and (3) frequently held by their claws. During handling the sloth’s behaviour was also analysed; we found that the two behaviours performed for the longest average duration of time were surveillance (55.3%) and limb stretching (12.6%). Further validation is needed; however, it is possible that some of the behaviours displayed may be indicators of fear, stress and anxiety. Our results describe behaviours exhibited by sloths, which have previously never been documented in any published literature, the data therefore serves as a potential baseline for future study.

**Abstract:**

The use of wild animals as photo props is prevalent across the globe and is widely recognised to represent a potential animal welfare concern. However, detailed information regarding the specific impacts of such activity on wild animal behaviour is currently lacking. Herein, we investigated how brown-throated three-toed sloths (*Bradypus variegatus*) were handled by tourists, and how sloths behaved during wildlife ‘selfies’ taken in Manaus, Brazil and Puerto Alegria and Iquitos in Peru. In total, we observed 17 sloths (during 70 focal observations) that were provided for use in wildlife selfies on 34 different tours. We found that an average number of 5 people held each sloth during each focal observation. For 48.6% of the time the sloths were handled in a way which involved physical manipulation of the sloths’ head and/or limbs and/or being held by the claws. From the eight different types of sloth behaviour observed, we found that the two types performed for the longest average duration of time were surveillance (55.3%) and limb stretching (12.6%). Our findings show that when being handled sloths were frequently held in ways that may compromise their welfare. Although to date the behaviour of sloths while being handled has not been reported in any published literature, in this study we document certain behaviours which may act as indicators of compromised welfare. We suggest that our data provides a potential baseline for future study into the behaviour and welfare of sloths.

## 1. Introduction

### 1.1. Wildlife Tourism 

Globally, there is a huge demand for wildlife tourism, and it’s likely to continue to increase in the future [1]. Wildlife tourism consists of a variety of activities, including observing animals in their natural habitats, viewing or interacting with animals in captivity, and fishing and hunting [2]. Wildlife tourism can have intended mortalities, for example in the case of fishing and hunting, but many types do not involve intended fatal outcomes by tourists [2]. The impacts of such activities on the welfare of the individual animals involved vary widely and can be positive, neutral or negative [3,4]. For example, research has found that the presence of tourists causes stress and anxiety in non-captive Barbary macaques [5]; and has a negative effect on the normal behavioural repertoire of dolphins and turtles [6,7], affects fledgling weights of yellow-eyed penguins [8] and increases the frequency of alert, fear, stress and aggressive behaviours in Asian elephants [9]. Within zoos, visitor presence has been reported to have a negative impact on the welfare of a range of species including, koalas [10], European red squirrels [11], gorillas [12,13] and spider monkeys [14].

### 1.2. Physical Contact 

Tourist attractions involving direct physical contact with wild animals are prevalent and are increasingly becoming a cause of animal welfare concern [15,16]. In one study it has been reported that 249 TripAdvisor webpages are currently advertising tourist attractions that advertise direct contact with wild animals [17]. Combined with poor husbandry and unnatural surroundings, close physical contact with wild animals can lead to stress, disease, injury and death [18]. One study, which explored the behavioural and physiological effects of handling on armadillos, hedgehogs and red-tailed hawks, found that animals which were handled for longer displayed higher levels of undesirable and self-directed behaviours [19]. Furthermore, fecal glucocorticoid metabolite levels increased when frequency and duration of handling increased [19].

One specific type of tourist activity includes using captive wild (i.e., non-domesticated) animals as photo props, where animals are used by tourists to pose with for photographic souvenirs [20,21,22]. Capturing one’s experience in a photo is becoming ever more important for distribution on social networking sites, it has been reported that today’s culture of having souvenir photographs for encounters with wildlife may be a factor which is contributing to the illegal trade of animals to be used as photo props [17]. For example, in recent years there has been an increase in the illegal trade of slow lorises out of Asia for the photo prop and pet trade industry [21]. From 2012–2013, 67 slow lorises were removed from the streets of Phuket [21]. Of the ten individuals examined six had their teeth clipped, to make them less sharp, such activity has devastating effects on the welfare of the animals involved, which inhibits them from being released back into the wild, and often results in their early death [21]. When photo prop tourism involves the repeated long term removal of individuals from the wild, this potentially could affect wild population numbers [17].

### 1.3. Measuring Handling Stress

When wild animals are handled, for example when being used as photo props, the experience can be physically and psychologically stressful for the animals involved [18]. This is often demonstrated through their physiology and behaviour [23]. However, measuring the effects that handling has on animals is complex and challenging [23,24]. This is partly because the term handling refers to a variety of different procedures, for example moving animals from one place to another, restraining them, or simply just holding them [23].

To accurately assess an individual animal’s reactions, a combination of reliable and validated behavioural and physiological measurements are most effective [24]. Although not explored in this study, physiological indicators of emotional and physical stress may include measurements of ventilation rates, cortisol levels and heart rate [25]. More recently, non-invasive measures (measures not involving penetration of the body for example, by incision or injection) are being explored as indicators of emotional states, for example nasal temperatures, specific behavioural indicators such as ear postures and percentage of visible eye whites [26,27,28]. In relation to stress levels associated with handling, hedgehogs, hawks and armadillos, have been assessed by extracting glucocorticoid metabolites from fecal samples [19]. Non-invasive measures are beneficial, as in many instances restraining animals, for example to take a blood sample, can cause stress, resulting in inaccurate assessments [26,28,29].

There are various reported negative and positive effects of handling on both domesticated and wild animals. Negative human-animal interactions, for example when animals are held inappropriately, can increase fearfulness in animals [30]. Negative handling that includes shouting and hitting have been shown to reduce welfare in cattle [31]. In wild animals, during translocation a single incident where an un-habituated animal is captured, handled and then re-released can result in a stress response, with lasting effects, which can negatively impair the animal’s physical and psychological welfare [32].

In contrast, in some instances handling has been associated with environmental enrichment and has a positive experience for some wild animals which are habituated to the presence of humans [23,33] Benefits of gentle handling and touching have been demonstrated in domestic species, including reduced levels of anxiety in dairy cows [34].

### 1.4. Sloths

It has been reported that annually hundreds of two and three-toed sloths are taken from deforested areas in Colombia and Brazil, where they are illegally traded for the pet trade and also used as food [35]. Often young two and three- toed sloths are taken from their mothers and exposed to inadequate housing and care [35]. The stress of capture and the captive conditions leads to high mortality rates, mother sloths are also often killed [35]. In addition to being used in the pet trade and for food, our own preliminary observations indicate that sloths are being used in wildlife tourism as photo props [22]. However, the number of sloths poached for this purpose, and the effect this activity has on their behaviour and welfare has not yet been assessed. Therefore, the aim of this research is to assess how brown-throated three-toed sloths (*Bradypus variegatus*) are handled during ‘selfies’, and explore how sloths behave during this type of tourist activity. Conclusions on the potential welfare implications will be drawn.

## 2. Methodology

### 2.1. Ethics

The research involved observations of sloth focused tourism activities that were already in existence, therefore ethical approval was not required.

### 2.2. Subjects and Study Locations

The study species was the brown-throated three-toed sloth (*Bradypus variegatus).* During the study 17 sloths were observed (9 adults and 8 juveniles). All sloths were wild caught, therefore further background histories, including their precise ages, and information about how and when they were taken from the wild could not be obtained. We conducted our study at three locations, these sites were where tourists could handle sloths. These sites are in Manaus in Brazil, and Puerto Alegria and Iquitos, both in Peru (See Table 1 for number of sloths and focal observations, and the total observation length at each location). At these sites, the sloths were handled by tour guides, who openly offered the sloths to tourists to handle on floating platforms, where souvenirs were also being sold. The housing conditions of the sloths when not being handled was not assessed during the study. Two researchers attended tours over the course of 34 days (1 tour per day) between 4 October 2016 and 8 April 2017. These tours were organised by 25 tour operators. We booked tours in advance, with key selection criteria being that operators verbally promoted the opportunity to have direct physical contact with wildlife. 

### 2.3. Procedure and Data Collection

**Figure 1:**
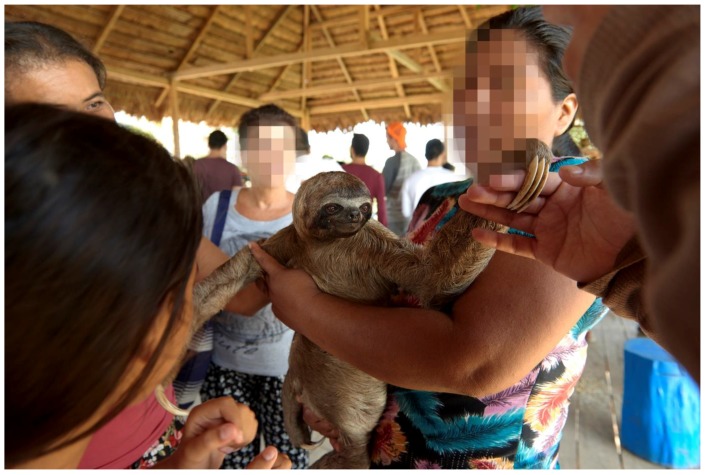
Platform in Puerto Alegria, where tourists are offered direct contact with sloths. Photograph taken by Fernando Carniel Machado.

On separate days two researchers attended the tours as tourists. The tours took place between 10:00 a.m.–17:00 p.m. Each tour followed a similar pattern, during the first part of the tour tourists congregated on a platform typically for one hour. In addition to handling brown-throated three-toed sloths, tourists were offered the opportunity to hold other species including common caiman, green anaconda and to touch free-ranging baited squirrel monkeys, various parrot species and toucans. As soon as this part of the tour began, the researchers started filming (using GoPro HERO4 Session and Sony HDR-CX625 cameras, GoPro, Inc., San Mateo, CA, USA). The researchers stood 0.5–2 meters from sloths being handled by the tourists or tour guides and filmed continuously for the entire handling period (including when the sloth was passed between people), this was considered one focal observation (handling refers to when tourists or tour guides were physically holding one or more sloth(s).) When tourists held the sloths, they were often not supervised (Figure 1).

Filming stopped when the focal sloth went out of sight, and a new focal observation started when there was an opportunity to film another sloth being handled. Each sloth was given a name to help aid identification, the sloths were identified by their physical characteristics, including size and fur patterns. The handling period varied in length, resulting in the focal observations being of various lengths. Also, the number of focal observations per animal varied, as some sloths were present on more of the tours attended than others. At the end of each day the video footage was uploaded to a server and backed up to be analysed later. 

### 2.4. Video Analyses 

From preliminary observations across all study sites, an ethogram was developed (Table 2). Each of the focal observations attained at the tourist sites were analysed by two researchers (the researchers were different to those who obtained the video footage) to determine the duration of the behaviours listed in the ethogram. If the sloths became out of sight at any point during the observations this was recorded. This allowed the duration of time each sloth was in sight for each observation to be determined. In addition to coding the duration of the observed behaviours, information about how the sloths were handled was also recorded during each observation. 

Firstly, the number of handlers during each observation was recorded. This included the number of individual people (both tourists and tour guides) that held the focal sloth. The number of passes between people was recorded. This was defined as the number of times each sloth was passed back and forth between handlers, for example the same person may have held the focal sloth twice. A handling score was also given to each focal observation (Table 3). A score of 0–3 was given to each observation, based on the definitions in Table 3. This score was an average score based on all the handling that each focal sloth had experienced during each observation. The handling scores were developed based on recommendations made by a member of the group of specialists of the International Union for Conservation of Nature/Species Survival Commission (IUCN/SSC) Anteater, Sloth and Armadillo Specialist Group (ASASG) on best practice when handling sloths. We considered direct physical manipulation of the body, limbs and/or head to contribute to poor handling. As sloths are arboreal, being supported when being handled is considered important (Table 3). We performed regular inter and intra-observer tests. The results of these reached a minimum of 95% agreement.

### 2.5. Data Analyses

For the behavioural data, descriptive statistics are reported. We first calculated the percentage of time that each sloth performed each behaviour for each focal observation. Using this data, we calculated an average percentage (median) for combined focal observations for each sloth (Supplementary Table S1). We also calculated the average (mean) percentage of time spent performing each behaviour for all sloths combined.

We used SPSS Statistics Version 22 (IBM SPSS Statistics, Armonk, NY, USA) to perform the statistical analyses. Data was assessed for normality. The data did not meet this assumption; therefore, non-parametric statistical tests were conducted. The duration of time engaged in each behaviour (while the sloths were in view) were analysed using the Friedman test for related samples. This was used to identify if there was a significant difference between the duration of time spent performing each behaviour. 

In relation to the handling data, we used descriptive statistics to summarise the data. The mean number of handlers and the mean number of passes between handlers was calculated for each of the three sites, and also combined for all the sites. Regarding the handling scores, a total for each of the four scores was calculated for each location, and combined for all three sites.

## 3. Results

We found that tourists had the opportunity to handle sloths on tours organised by 19 of the 25 tour operators (76%). At the tourist sites 70 focal observations were completed in total across the three locations, comprising of 233 min in duration in total during the study period. The focal observations ranged in duration from 8 s to 11 min. This was determined by how long the tourists wanted to hold each sloth for, and by how busy the tour was (if the tours were busy then the sloths would be passed around at a quicker rate). The number of focal observations per animal ranged between 1–11 (*M* = 4.1). 

### 3.1.Tourists and Handling Scores 

During the 70 focal observations, the mean number of people that held the sloths was 5 (range 1–24). The mean number of times the sloths were passed between people was 6 (range 0–26). Regarding the four handling scores, for all three sites combined score 0 was the most common score (*n* = 34, 48.6%), followed by 1 (*n* = 17, 24.3%), 2 (*n* = 12, 17.1%) and 3 (*n* = 7, 10%). Table 4 shows that the average number of people handling the sloths and the handling scores were similar between the tourist sites.

### 3.2. Sloth Behaviour

**Figure 2:**
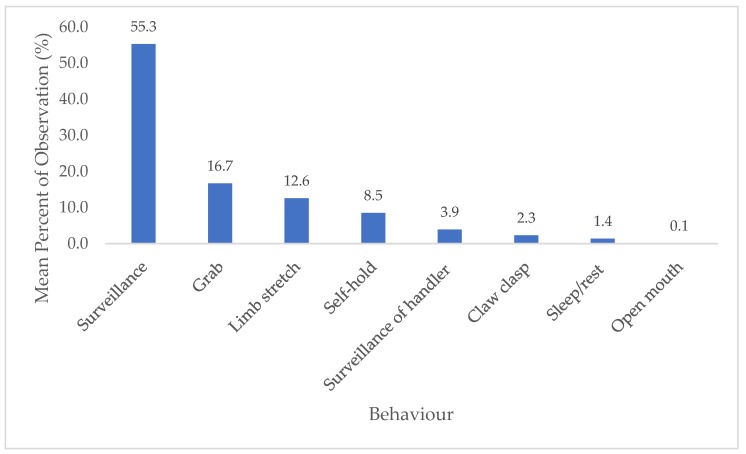
Mean percentage of time the sloths spent engaged in each behaviour.

During handling, the sloths were seen to engage in eight different types of behaviour. A significant difference was found between the duration of the behaviours performed (*Χ*^2^ = 68.078, *df* = 6, *p* = < 0.01). During the focal observations (for all sloths combined) we found that the two behaviours performed for the longest mean duration were surveillance (55.3%) and limb stretching (12.6%) (Figure 2).

The duration of time spent performing the eight behaviours were compared between adults (*n* = 9) and juveniles (*n* = 8) a significant difference was found for the behaviour surveillance of handler (*Χ*^2^ = 5.373, *df* = 1, *p* = < 0.05). With juveniles spending 6.1% of the total observation time observing their handler and adults spending 1.5% of their time engaged in this behaviour. In relation to the other behaviours no significant differences were found (*p* = > 0.05). 

## 4. Discussion

This is a preliminary study, which aimed to investigate how brown-throated three-toed sloths were handled during wildlife selfies at three different tourist locations in Latin America, and the impact that handling might be having on their behaviour. Our results show that sloths were held repeatedly by numerous tourists, often being unsupported and/or having their limbs or head physically manipulated. It’s likely that tourists engaging in this activity are not aware of the potential negative impact that handling has on the behaviour and welfare of sloths. Although further research is needed, our study also shows that when being handled the sloths performed behaviours which have not been reported in any published studies looking at the behavioural repertoire of sloths in a natural environment. Behaviours such as surveillance and sleep/rest, which have been described in sloths in the wild were found to be performed at different rates during this study. 

### 4.1. Handling Scores

Score 0 was the most common handling score given, followed by score 1. Score 0 was given to a handling period when the sloth’s heads or limbs were manipulated and/or they were held only by their claws. It is highly likely that physical manipulation such as this would be stressful for the sloths involved, furthermore when sloths are held from their claws they are left completely unsupported. Score 1 meant that for 50% of the time or more the sloths had limited opportunity to cling onto the handler. Being arboreal mammals, when sloths are handled they need to have support that provides security, in their natural environment branches would provide support and transportation through the forest [35].

### 4.2. Surveillance Behaviour

Our results show that when sloths were being handled at the three tourist sites they spent 55.3% of the focal observation periods surveying their surroundings. Currently there is no other published research which has assessed this behaviour in captive sloths (either wild-caught or captive-bred). However, there are some studies which have surveyed the behaviour of wild brown-throated three-toed sloths in their natural environment. During one study it was found that in the forest they spend approximately 10% of their time engaged in surveillance of their surroundings [36]. In a second study brown-throated three-toed sloths were observed to 17.3% of their time engaged in surveillance [37]. It is widely accepted that the primary function of vigilance in non-human mammals is to detect and avoid predators [38,39]. It has also been reported in other mammals that levels of vigilance can be used to measure fearfulness and anxiety [38] and that high levels of visual surveillance may reflect anticipation of danger [40]. Studies looking at visitor effects on a range of mammalian species housed in zoos have found increased levels of vigilance resulting from the presence of people. For example, in western lowland gorillas [12], orangutans [41] and koalas [10]. 

Increased vigilance has also been found in Asian rhinos inhabiting a national park, which was open to tourists [42]. In these examples, increased vigilance has been interpreted as potential signs of stress, anxiety and/or fear [10,12,41,42]. In line with research in other mammalian species, the increased level of vigilance observed could indicate that the sloths were experiencing fear and anxiety due to being in direct contact with people. However, as little is known about the behaviour repertoire of sloths when in contact with people we cannot rule out the possibility that the sloths were curious of their surroundings.

In addition to surveillance of surroundings, data on surveillance of the handler in the tourist setting was gathered. A significant difference was found between adults and juveniles in the performance of this behaviour, it is unclear as to why this is. However, this highlights the need for further investigation into the differences between juveniles and adults.

### 4.3. Additional Behaviours

In the wild brown-throated three-toed sloths have been observed to spend an average of 56% of their time sleeping/resting [36]. When being handled by people the sloths only engaged in this behaviour for 1.4% of the time. During each focal observation, the sloths were held on average by 5 people, and the mean number of times they were passed between people was 6. It is possible that the large turnover of handlers combined with the high levels of surveillance observed resulted in the sloths not having the opportunity to rest or sleep when being handled. In relation to the implications of this on the sloth’s welfare, as the behaviour of the sloths were not observed during periods outside of being handled, it cannot be determined in this study if they had opportunities to sleep or rest outside of the handling periods. If the sloths were deprived the opportunity to sleep/rest outside of the handling periods, then it is likely that this would have serious negative impacts on their welfare. There were some additional behaviours which have not been described in any published literature, these were self-hold, claw clasp and limb stretch. It is possible that these behaviours represent displacement or self-directed behaviours, indicating stress and anxiety. However, more research is needed in different environments to validate and understand the behaviours further. Self- directed/displacement behaviours are considered behavioural indicators of stress and anxiety in a range of species both in captivity and in the wild [43]. For example, in non-human primates self-scratching, self-grooming, self-touching, body shaking and yawning have been reported to increase during anxiety inducing situations [43,44,45]. 

When being handled, the sloths were observed to ‘grab’ people 9.7% of the time. There are several reasons which may explain why this behaviour occurred. The high number of tourists holding the sloths may have been a contributor. It could be that the ‘grabbing behaviour’ represents the sloths clinging/holding on to different people when they were passed around. Also, they may not have been comfortable or secure in how they were being handled. This potentially could also have been an aggressive display, however as this behaviour has not been described in the scientific literature further work is needed to explore this behaviour

### 4.4. Limitations and Future Research

In addition to analysing the sloth’s behaviour, it would have been beneficial to gather physiological data such as glucocorticoid samples, which in addition to behaviour can give an insight into an animals well-being [46]. This could be particularly beneficial in species with a slow metabolism, resulting in a restricted behaviour repertoire, such as sloth species. Such data may have provided further support for stress and fear in the sloths. However, due to the experimental design and the ‘opportunistic’ data collection method (the study was not a controlled experiment) it was not logistically possible in this study to collect physiological data. It is also worth noting that some techniques for collecting glucocorticoid data are invasive, and could have affected the analysis of the effects of handling, this is a common problem of assessing stress in wild animals [46]. 

Furthermore, it may be useful to consider noise levels in future studies, as this may have had an impact on the sloths’ behaviour. It is possible that the background histories of the sloths, their personalities and their past experiences with people, including how and at what age they were taken from the wild may also affect their behaviour. This is evident in the differences observed between the sloths in regards to the average duration of time they engaged in each behaviour (Supplementary Table S1).

As the animals were illegally traded it was not possible in this study to obtain this information but future studies exploring sloth behaviour should consider these factors. As this is the first study describing the behaviour of sloths during periods of potential stress, the interpretation of our data needs further validation from studies exploring the behaviour of sloths in different environments. In this study there wasn’t a control situation, further research could address this by comparing the behaviour of sloths between periods of handling and non-handling within the same context. We acknowledge that our sample size was small, which provided limited options for statistical analysis, this is reported as is a common problem in behavioural studies [47]. Future studies could aim to increase sample sizes, focal observation lengths, and the number of focal observations per animal, however this is not always possible particularly with elusive species. 

The behaviour of adults and juveniles was compared, a significant difference was found in the duration of time spent engaged in surveillance of handler. Future studies should continue to explore differences between adults and juveniles, as it may be possible that the age at which they are taken from the wild and subjected to direct contact with people effects their behavioural responses and coping mechanisms. Also adults, juveniles and infants have been reported to have different behavioural repertoires in the wild [36]. In this study the sex of the sloths could not be determined, but future studies exploring the behaviour of captive sloths should compare the behaviour of males and females. 

## 5. Conclusions

Our study builds on existing studies which highlight the animal welfare concerns associated with direct physical contact between tourists and wildlife across Latin America [17,22]. The study demonstrates that sloths being used for selfies are subjected to handling which likely compromises their welfare, with their heads and limbs being manipulated. Furthermore, they behave in a way which may indicate fear and stress. Results from this study certainly warrant further investigation into the impact that this type of direct physical contact has on the behaviour and welfare of sloths. As suggested by previous studies [17], more research focused on the attitudes of tourists is required. Such research will help to inform public awareness and education initiatives aimed at reducing the demand for ecotourism which may negatively impact the welfare of wildlife [22].

## Figures and Tables

**Table 1:** Number of sloths, focal observations, and total observation length at each location.

Location	Number of Sloths at Each Location	Number of Focal Observations at Each Location	Total Observation Length at Each Location (Minutes)
Manaus, Brazil	9	45	161.7
Puerto Alegria, Peru	4	11	31.3
Iquitos, Peru	4	14	40.1

**Table 2:** Ethogram of behaviours recorded.

Sloth Behaviour	Description of Sloth Behaviour
Sleep/rest	Body motionless, subject does not appear to be vigilant, eyes may be open or closed.
Self-groom	Scraping the surface of the fur in a back and forth motion making use of the claws.
Eating	Ingestion of edible material.
Movement	Movement of limbs to move from one location to another.
Limb stretch	A full extension of the arms and/or legs. They are deliberately held out and extended.
Surveillance of handler	Eyes are open and the sloth’s head moves left and right following the motion of the handler and/or makes eye contact clearly demonstrating that the handler has been perceived.
Surveillance	Eyes are open and the sloth’s head moves left and right clearly observing their surroundings.
Grab	Contact or attempted contact of foot or hand with a person. Ends when the hand or feet claws move back to their original position.
Defecation/urination	Defecates or urinates
Claw clasp	Claws are held together (either hands or feet) and held out in front of the body.
Self-hold	A limb is held for example, hand holds foot.
Open mouth	Mouth is held open

**Table 3:** Handling scores and descriptions.

Handling Score	Description of Handling
0	The sloth’s limbs or head are manipulated in some way and/or held in a certain position on one or more occasions. and/or The sloth’s is held by his/her claws on one or more occasion(s)
1	For 50% of the time or more the sloth is held around the chest and is facing away from the handler or towards the handler but they are un-supported. Their legs and arms are left hanging. They have no or a limited opportunity to hold or cling onto the handler.
2	Intermediate between 1 and 3. For less than 50% of the time the sloth is held around the chest and facing away from the handler. In some instances, they are fully supported, facing inwards towards the handler.
3	The sloth’s body is fully supported by most the handlers for 80% of the time or more. The sloth is held facing in wards towards the handler’s body. The sloth can cling/hold on to the handler.

**Table 4:** Mean number of handlers and handling score for each tourist site.

Location	Mean Number of Handlers	Mean Number of Passes	Mean Handling Score
Manaus, Brazil	5	6	1
Iquitos, Peru	4	4	0
Puerto Alegria, Peru	5	5	1

## Supplementary Materials

The following are available online at http://www.mdpi.com/2076-2615/8/11/216/s1, Table S1: Median duration (percentage) for each behaviour performed, and percentage ranges for each sloth.
