# Supplementary material for: The Impact of ‘Selfie’ Tourism on the Behaviour and Welfare of Brown-Throated Three-Toed Sloths
Source: Animals (Basel). 2018 Nov 19;8(11):216. doi: 10.3390/ani8110216 (PMC6262464; doi:10.3390/ani8110216)
Supplement: Supplementary file 1 [file animals-08-00216-s001.pdf]

animals

Supplementary Materials: The Impact of 'Selfie' Tourism on the Behaviour and Welfare of Brown–Throated Three-Toed Sloths

Gemma Carder [1,*], Tinka Plese [2], Fernando Carniel Machado [1], Suzanne Paterson [1], Neil Matthews [1], Laura McAnea [1] and Neil D'Cruze [1]

[1] World Animal Protection, London, WC1X 8HB, UK; icenando@gmail.com (F.C.M.); suzipaterson@hotmail.co.uk (S.P.); neilsmatt2@gmail.com (N.M.); lmcanea@gmail.com (L.M.A); neildcruze@worldanimalprotection.org (N.D.C)

[2] Aiunau Foundation, Circular 1ª, No. 73 — 20. Medellín, Colombia, South America; f.aiunau@gmail.com

* Correspondence: gemmacarder@yahoo.co.uk; Tel.: +44–0–779891551

Received: 30 August 2018; Accepted: 3 November 2018; Published: 19 November 2018

Supplementary table 1. Median duration (percentage) for each behaviour performed, and percentage ranges for each sloth.

Sloth No	No of focal observations	Duration of Behaviours							
		Sleep/ rest	Limb stretch	Surveil–lance of handler	Surveilla–nce	Grab	Self –hold	Claw clasp	Open mouth
1	7	*M*= 0 r= (0–29)	*M*= 0 r= (0–6.1)	*M*= 2.5 r= (0.3–6.9)	*M*= 2.5 r= (18.8–98.9)	M=5.6 r= (0.8–25.4)	*M*= 0.3 r= (0–77.4)	*M*= 0 r= (0–2.2)	*M*= 0 r= (0–2.2)
2	11	*M*= 0 r= (0–0)	*M*= 4.4 r= (0–31.8)	*M*= 4 r= (0–16.8)	*M*= 66.9 r= (15–92.9)	*M*= 14.2 r= (3.2–100)	*M*= 0 r= (0.7–1.9)	*M*= 0 r= (0–0)	*M*= 0 r= (0.2–0.7)
3	3	*M*= 0 r= (0–0)	*M*= 30.3 r= (12.5–62.6)	*M*= 0 r= (0–0.7)	*M*= 42.6 r= (42.6–70.6)	*M*= 3.7 r= (0–4.8)	*M*= 0 r= (0–0)	*M*= 28.2 r= (0–48.5)	*M*= 0.4 r= (0–0.8)
4	7	*M*= 0 r= (0–0)	*M*= 20.7 r= (0–65.9)	*M*= 8.3 r= (0–30.2)	*M*= 61.4 r= (20.2–97.9)	*M*= 4.3 r= (0–42.6)	*M*= 0 r= (0–61.7)	*M*= 0 r= (0–81.9)	*M*= 0 r= (0–0)
5	7	*M*= 0 r= (0–0)	*M*= 2.1 r= (0–4.2)	*M*= 0 r= (0–2.7)	*M*= 91.5 r= (38.8–100)	*M*= 14.7 r= (7–100)	*M*= 0.4 r= (0–46.6)	*M*= 0 r= (0–0)	*M*= 0 r= (0–0.06)
7	2	*M*= 0 r= (0–0)	*M*= 11.6 r= (3.9–19.3)	*M*= 0.7 r= (0–1.4)	*M*= 30.5 r= (19.3–41.7)	*M*= 9.2 r= (6.5–11.9)	*M*= 27.2 r= (4.6–49.7)	*M*= 0 r= (0–0)	*M*= 0 r= (0–0)
9	7	*M*= 0 r= (0–0)	*M*= 0 r= (0–0)	*M*= 0 r= (0–29.7)	*M*= 56.3 r= (30.5–100)	*M*= 7.7 r= (2.6–21.2)	*M*= 0 r= (0–0)	*M*= 0 r= (0–0)	*M*= 0 r= (0–0.5)
11	6	*M*= 0 r= (0–0)	*M*= 0 r= (0–28.6)	*M*= 0 r= (0–7.9)	*M*= 59.3 r= (0–100)	*M*= 1.6 r= (3.2–13.4)	*M*= 0 r= (0–0)	*M*= 0 r= (0–0)	*M*= 0 r= (0–0)
12	10	*M*= 0 r= (0–66.3)	*M*= 3.4 r= (0–100)	*M*= 1.6 r= (0–13)	*M*= 30.8 r= (5.8–51.6)	*M*= 3.2 r= (0–10.7)	*M*= 2.3 r= (0–10.7)	*M*= 0 r= (0–0)	*M*= 0 r= (0–1.4)
13	2	*M*= 0 r= (0–0)	*M*= 13.1 r= (9.2–17)	*M*= 3.9 r= (0–7.9)	*M*= 75.5 r= (65.8–85.2)	*M*= 7.8 r= (5.1–10.5)	*M*= 0 r= (0–0)	*M*= 0 r= (0–0)	*M*= 0 r= (0–0)
17	2	*M*= 0 r= (0–0)	*M*= 17 r= (6.7–27.3)	*M*= 1.3 r= (1.3–1.3	*M*= 56.3 r= (35–77.7)	*M*= 8.9 r= (6.1–11.8)	*M*= 0.7 r= (0–1.4)	*M*= 0 r= (0–0)	*M*= 0 r= (0–0)

* M= Median percentage, r= the percentage range. Sloths 6, 8, 10 and 14–16 are removed from this table as only one focal observation was possible with each of these sloths.
